# Clinical Protection from Falciparum Malaria Correlates with Neutrophil Respiratory Bursts Induced by Merozoites Opsonized with Human Serum Antibodies

**DOI:** 10.1371/journal.pone.0009871

**Published:** 2010-03-25

**Authors:** Charlotte Joos, Laurence Marrama, Hannah E. J. Polson, Sandra Corre, Antoine-Marie Diatta, Babacar Diouf, Jean-François Trape, Adama Tall, Shirley Longacre, Ronald Perraut

**Affiliations:** 1 Institut Pasteur de Dakar, Unité d'Immunologie, Dakar, Sénégal; 2 Institut Pasteur, CNRS, URA 1961, Laboratoire de Vaccinologie Parasitaire, Paris, France; 3 Institut Pasteur de Dakar, Unité d'Epidémiologie, Dakar, Sénégal; 4 IRD, Laboratoire de Paludologie/Zoologie Médicale, Dakar, Sénégal; Walter and Eliza Hall Institute of Medical Research, Australia

## Abstract

**Background:**

Effective vaccines to combat malaria are urgently needed, but have proved elusive in the absence of validated correlates of natural immunity. Repeated blood stage infections induce antibodies considered to be the main arbiters of protection from pathology, but their essential functions have remained speculative.

**Methodology/Principal Findings:**

This study evaluated antibody dependent respiratory burst (ADRB) activity in polymorphonuclear neutrophils (PMN) induced by *Plasmodium falciparum* merozoites and antibodies in the sera of two different African endemic populations, and investigated its association with naturally acquired clinical protection. Respiratory bursts by freshly isolated PMN were quantified by chemiluminescence readout in the presence of isoluminol, which preferentially detects extra-cellular reactive oxygen species (ROS). Using a standardized, high throughput protocol, 230 sera were analyzed from individuals of all age groups living in meso- (Ndiop) or holo-endemic (Dielmo) Senegalese villages, and enrolled in a cross-sectional prospective study with intensive follow-up. Statistical significance was determined using non-parametric tests and Poisson regression models. The most important finding was that PMN ADRB activity was correlated with acquired clinical protection from malaria in both high and low transmission areas (*P* = 0.006 and 0.036 respectively). Strikingly, individuals in Dielmo with dichotomized high ADRB indexes were seventeen fold less susceptible to malaria attacks (*P* = 0.006). Complementary results showed that ADRB activity was (i) dependent on intact merozoites and IgG opsonins, but not parasitized erythrocytes, or complement, (ii) correlated with merozoite specific cytophilic IgG1 and IgG3 antibody titers (*P*<0.001 for both), and (iii) stronger in antisera from a holo-endemic compared to a meso-endemic site (*P* = 0.002), and reduced in asymptomatic carriers (*P*<0.001).

**Conclusions/Significance:**

This work presents the first clearly demonstrated functional antibody immune correlate of clinical protection from *Plasmodium falciparum* malaria, and begs the question regarding the importance of ADRB by PMN for immune protection against malaria i*n vivo*.

## Introduction

There are 300–500 million clinical malaria cases annually, and 1–3 million deaths of primarily young African children [1]. Blood stage *Plasmodium falciparum* causes the most morbidity and mortality, and widespread multi-drug resistance has accentuated the need for an effective vaccine. Merozoite surface proteins (MSPs) are considered relevant vaccine targets, because they mediate erythrocyte invasion, and are accessible to antibodies and complement. Indeed, high IgG antibody titers to some MSP recombinant antigens have been correlated with acquired immunity in endemic populations [2]–[5]. One postulated antibody function is to inhibit parasite growth by preventing erythrocyte invasion and/or by interfering with intra-erythrocytic development. The currently most widely accepted functional assays for antibodies to blood stage *P. falciparum* antigens measure inhibition of erythrocyte invasion and/or parasite growth *in vitro* [6]–[11], but the relevance of these assays to clinical protection in humans has never been clearly established.

A second category of antibody activity concerns antibody dependent cellular functions. Possible cellular immune effectors for blood stage malaria include polymorphonuclear neutrophils (PMN) and/or monocytes, since mature erythrocytes lack histo-compatibility antigens, and macrophages are found only in extra-vascular tissues. It has been proposed that monocytes activated by opsonized merozoites, release an unidentified soluble factor that inhibits the growth of surrounding intra-erythrocytic parasites. A functional antibody assay is based on this notion (antibody dependent cellular inhibition, ADCI) [6], [12], but it has not been widely reproduced in other laboratories, and the relevance of the assay itself to clinical protection in humans has not been statistically validated either in prospective longitudinal studies or otherwise.

Neutrophils are quite plausible immune effectors for the control of *Plasmodium* blood stage infection because they are the most numerous blood leucocytes (50–75%), and the best circulatory phagocytes. Opsonized merozoites are known to be phagocytosed by neutrophils *in vitro* [13], [14] and *in vivo* [15]. In addition, neutrophils are particularly effective generators of reactive oxygen species (ROS), which are highly toxic for intra-erythrocytic malaria parasites [12], [16]–[18] and high ROS production by neutrophils has been correlated with fast parasite clearance in Gabonese children with *P. falciparum* malaria [19].

These observations suggested that ROS release by activated neutrophils might be more involved in immune protection from malaria than generally appreciated. Surprisingly, reviews of immune effector mechanisms against blood stage *Plasmodium* infection rarely mention or highlight roles for neutrophils or ROS [20], [21]. Neutrophil respiratory bursts can be quantified by emitted light detected with chemiluminescence dyes [22]–[25], and can be triggered by opsonized *P. falciparum* merozoites, and enhanced by cytokines [26]. Large scale functional analysis of anti-merozoite antibodies using this approach, has been limited by a lack of miniaturized and/or automated procedures [13], [26]–[28]. To investigate relationships between neutrophil antibody dependent respiratory burst (ADRB) activity and acquisition of natural immunity to malaria, a reproducible, standardized, high throughput *in vitro* chemiluminescence protocol was developed, based on merozoites opsonized with antibodies in the sera of two *P. falciparum* exposed populations. The results presented constitute the first demonstration of a functional antibody correlate of clinical protection against *P. falciparum* malaria. The ADRB assay could help to reliably prioritize MSP vaccine candidates [8], [29]–[31] for clinical testing, and its surprising statistical correlations may focus new attention on the *in vivo* relevance of the ADRB mechanism.

## Materials and Methods

### Study sites

Two villages in Senegal with different malaria epidemiology were studied. In Dielmo malaria transmission is perennial because a nearby river maintains continuous mosquito breeding sites. In Ndiop, transmission is strictly seasonal and depends on the rainy season that normally occurs from July to September. This differing transmission is reflected in relatively constant *P. falciparum* prevalence rates of >80% in Dielmo compared to seasonal rates in Ndiop that change from 20% in the dry season to 70% in the rainy season [32]. Consequently, the peak incidence of clinical malaria in Dielmo occurs in very young children of 2–5 years and declines rapidly with age, whereas in Ndiop peak incidence is in children of 5–10 years with only a gradual decrease in age. A field research station with a dispensary was built for the project in each village to identify and treat all episodes of morbidity.

### Subjects and ethics statement

The Dielmo and Ndiop longitudinal surveys have been described in detail elsewhere [32], [33]. The project protocol and objectives were carefully explained to the assembled village population and informed consent was individually obtained from all subjects either by signature or by thumbprint on a voluntary consent form written in both French and in Wolof, the local language. Such consent was obtained in the presence of the school director, an independent witness. For very young children, parents or designated tutors signed on their behalf. The protocol and consent procedure was approved by the Ethical Committee of the Institut Pasteur de Dakar and the Ministère de la Santé of Senegal. An agreement between Institut Pasteur de Dakar, Institut de Recherche pour le Développement (IRD) and the Ministère de la Santé et de la Prévention of Senegal defines all research activities in Dielmo and Ndiop. Each year, the project was re-examined by the Conseil de Perfectionnement de l'Institut Pasteur de Dakar and the assembled village population; informed consent was individually renewed from all subjects.

In order to comprehensively identify all episodes of morbidity, villagers were under constant daily medical surveillance. At least one physician, a technician or a nurse, and three medical field workers were present in the village 24 hr a day, seven days a week. Active case detection was carried out such that each villager received daily home visits. For each clinical episode with a history of fever (≥38°C), headache, or vomiting, thick blood films were made for treatment decisions, and clinical symptoms were systematically recorded.

In July 2002, before the transmission season in Ndiop, villagers from Ndiop (114) and Dielmo (116) were enrolled in a longitudinal and cross-sectional study [32], [34]. None were symptomatic for malaria, but were not screened for circulating parasites (asymptomatic malaria). The mean age of the Ndiop and Dielmo cohorts was 24.0 (3.4–76.9) and 28.6 (3.4–80.5) years respectively. The number of infective bites per person per day during the period just preceding the beginning of the study was 0 and 1.97 for Ndiop and Dielmo residents respectively. During the study period residents of Ndiop and Dielmo received an average of around 20 or 295 infective bites per inhabitant respectively (Boudin et al., unpublished data). After venous puncture, RBCs were removed by centrifugation and plasma was stored at −20°C.

### Antibodies

Sera were de-complemented by heating 20 min at 56°C. A hyper-immune serum pool (HIS) from 30 adult residents of Dielmo, and non-immune serum pool (NIS) obtained commercially (Valbiotech, France), were the positive and negative controls respectively. Total IgG was purified using Ultralink® immobilized Protein-G (Pierce) according to the manufacturer's instructions. Briefly, sera (0.4 mL) were diluted 1∶5 in binding buffer, passed 3 times over 2 mL of packed resin and washed with 15 column volumes of binding buffer. IgG was eluted with 10 mL of elution buffer, neutralized with 1M Tris pH 8.0, dialyzed against PBS, and concentrated to 0.4 mL (Amicon Ultra, 5,000 MWCO; Millipore). IgG-depleted sera were obtained by concentrating flow-through fractions to the initial 0.4 mL volume.

High titer rabbit polyclonal anti-PfMSP1p19 antibodies raised to purified baculovirus recombinant PfMSP1p19 [35], were used to characterize merozoite preparations.

### Parasite culture and merozoite preparation

Palo Alto (FCR3) *P. falciparum* was cultured continuously on O^+^ erythrocytes in RPMI containing 0.5% Albumax and 1 µg.mL^−1^ gentamycin, in candle jars [36]. Merozoite extracts were prepared by two methods. (i) Cultures with >5% parasitemia were centrifuged 5 min at 400xg. RBCs (pellet) were replaced in culture, and merozoites in supernatants were recovered by centrifugation for 20 min at 1500xg and stored at −20°C in the RPMI/Albumax culture medium. (ii) After synchronization using D-sorbitol, cultures containing mature schizonts were centrifuged 10 min at 700xg at RT on a 75% isotonic percoll cushion. Merozoites in the RPMI-percoll interface were washed once in PBS, while infected erythrocytes in the pellet were washed in RPMI and replaced in culture. Merozoite pellets were stored at −20°C with no additives and aliquots were pooled as needed. Alternatively, sonicated merozoites, normal or parasitized RBC, or freeze-thawed parasitized RBC pellets were used for controls.

### Effector cell preparation

Blood samples from healthy adults presenting for routine blood tests at the Pasteur Institute, Dakar were obtained with informed written consent approved by the Ethical Committee of the Institut Pasteur de Dakar. Samples from 6–7 donors (4 mL each), were collected into EDTA-K3 tubes, layered onto Ficoll-Hystopaque (density 1.077, Sigma) and centrifuged at RT for 30 min at 400xg. PMN were harvested at the ficoll-RBC interface. Residual RBCs were lysed by incubation in 8.32 g.L^−1^ NH_4_Cl, 0.8 g.L^−1^ sodium bicarbonate, and 0.043 g.L^−1^ EDTA 8 min at 4°C and centrifuged at 400xg for 5 min. Purified effector cells were washed twice with Hank's balanced salt solution (HBSS), enumerated using trypan blue, and resuspended in PBS at 1–5.10^7^ cells mL^−1^, depending on the quantity of PMN collected on a given day and the number of wells tested. Within this concentration range there was no effect on readout under the conditions of the assay. The major contaminants were cellular debris and RBC. Viability (verified before every experiment) was >99% and PMNs constituted 90–99% of leucocytes. PMN were used immediately after harvest and purification, usually within 2–3 hr following sample collection.

### Chemiluminescence monitoring of ROS generation

Chemiluminescence was measured at 37°C using opaque 96-well plates (Berthold), and a MicroLumat Plus 96 luminometer (Berthold), controlled with WinGlow software. Chemiluminescence was recorded as relative light units (rlu). A 4 mg.mL^−1^ stock solution of isoluminol (4-aminophthalhydrazide; Sigma) prepared in DMSO, was stored in aliquots at –20°C in the dark, and used at a final concentration of 0.04 mg.mL^−1^ in sterile PBS. To facilitate rapid handling, only 40–50 wells per plate were used. Merozoite pellets (40 µL), or alternatively normal or parasitized red cell pellets, were incubated with 10 µL of test or control sera or IgG for at least 30 min at 37°C. PMN (100 µL at 1–5.10^7^ cells mL^−1^) and isoluminol (100 µL) were loaded rapidly using an Eppendorf Multipipette 4780. Plate reading started immediately, and each well was monitored automatically by the luminometer for 1 sec every minute for 1 hr. The parameter used for this work was maximal rlu, which was generally observed within minutes of initiating the reactions under the conditions used. For experiments using N-formyl-Met-Leu-Phe (fMLP), PMN were incubated with 20 µL per well of 2.5 mM fMLP (Sigma) at 37°C for 5 min before adding opsonized merozoites to start the reaction.

### Determination of the standardized ADRB index

The maximal rlu primary data were standardized using the HIS serum pool as the internal control. Due to relatively rapid maximal rlu readout, the HIS standard value was determined as the average of duplicate measurements in the first and last wells of every plate, although there was generally <5% difference in the two readings. Data are presented as a standardized merozoite Antibody Dependent Respiratory Burst (ADRB) activity index calculated as: 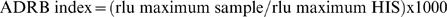



Only experiments in which the HIS rlu maximum was ≥100 (>6x background) were included in the analyses. An additional internal control with the same positive serum was included in each run.

### ELISA

Merozoites resuspended in PBS were lysed with 3 freeze-thaw cycles, dosed by BCA (Pierce), diluted to 10 µg.mL^-1^ in PBS and coated on MaxiSorp plates (Nunc). The ELISA protocol was described previously [3], [30]. The HIS positive control was titered on each plate in 2-fold dilutions starting from 1∶200 for IgG1 and IgG3, or from 1∶5,000 for total IgG. Samples were analyzed at 3 dilutions, either 1∶200, 1∶400 and 1∶800 for IgG1 and IgG3, or 1∶5,000, 1∶10,000 and 1∶20,000 for total IgG. Arbitrary titers were extrapolated from the HIS regression curve using a four-parameter logistic fit. The program (in Excel) constructs a sigmoid 4-parameter curve for the calibrator, by an interactive process. The unknowns are then calculated by the use of the 4-parameters estimated. The program can be obtained from Ed Remarque (remarque@bprc.nl), and is accompanied by a manual with all the formulae and estimation methods outlined.

### Statistical analysis

Antibody levels (ELISA analysis) and ADRB comparisons were analyzed for statistical significance using the Wilcoxon signed rank test and the Spearman rank correlation test for non-normally distributed data. The Mann Whitney test was used to compare unpaired data, and only *P*<0.05 was considered significant. A Poisson regression model was used to analyze the relationship between the ADRB index and the incidence of clinical episodes during the following transmission season (5.5 months in 2002) in Ndiop (mesoendemic), and during the same period in Dielmo (holoendemic).

Malaria episodes were defined as the presence of fever (≥38°C) and/or malaria-like symptoms with parasitemias higher than a given threshold value above which an individual had a significantly higher probability that the observed fever was due to malaria. Parasitemias were determined using thick smears where parasites and leucocytes, but not erythrocytes, can be reliably enumerated by Giemsa staining. Thresholds were determined statistically and have been used in other studies carried out in the two villages [3], [30]. For Ndiop the threshold was not age dependent, and was 30 trophozoites / 100 leucocytes (Christophe Rogier and André Spiegel, unpublished data). In Dielmo symptoms occurred as a function of age-dependent pyrogenic parasitological thresholds (Rogier et al. (1996) Am J Trop Med Hyg 54:613), which were the following (per 100 leucocytes): 0–12 months, 245 parasites; 12–23 months, 270 parasites; 2–5 yr, 240 parasites; 5–10 yr, 200 parasites; >10 yr, 155 parasites; >60 yr, 50 parasites. The same thresholds were used here, and didn't depend on conditions specific to the study. When fever persisted to the next day, another thick blood smear was made. Treatment was given to patients with fever and/or symptoms compatible with malaria if parasite density was above the threshold. This protocol and the intensive follow up of the study insured that febrile episodes were correctly categorized and that no malaria episodes were missed.

The time at risk for each villager was calculated as the number of days actually spent in the village during the 5.5-month follow-up period. Episodes were considered independent if separated by >15 days. Risk for each individual was reduced by 15 days following a diagnosis of malaria, or by 8–15 days following anti-malarial treatment regardless of diagnostic criteria, depending on the drug administered (8, 10, and 15 days for quinine, chloroquine, and sulfadoxine-pyrimethamine, respectively [37], [38]). The times used in this study are based on data concerning the half-life of the drugs used. Half-lives for quinine, sulfadoxine and pyrimethamine are respectively 10 hr, 8 days and 4 days [37]. For chloroquine a half-life of 10–70 hr was used (French notice on drugs and drug addiction / AFSSAPS; 13 December 2006). The intensive follow-up used in this study, including random urine tests, allowed us to determine precisely the beginning and the end of drug treatments and to eliminate any risk of auto-medication.

The Ndiop analysis included 114 individuals with a total of 121 clinical episodes during the follow-up period. The incidence of clinical malaria for each individual was calculated as the number of clinical episodes divided by the number of days at risk during the 5.5-month follow-up. The mean incidence as a function of age bracket, defined as the sum of individual incidences divided by the number of individuals in that age bracket, was: (i) 2–14 years: 0.015, n = 49 (ii) 15–29 years: 0.004, n = 32 and (iii) ≥30 years: 0.001, n = 33 (*P*<0.001). The Dielmo analysis included 116 individuals with a total of 50 clinical episodes during the follow-up period. The mean incidence of clinical malaria as a function of age bracket was (i) 2–6 years: 0.0188, n = 18, (ii) 7–14 years: 0.0018, n = 31 and (iii) ≥15 years: 0.0003, n = 67 (*P*<0.001). Age stratification was based on parasitological and clinical data obtained during a 10-year longitudinal study [3], [30], [32], [33], [39]. ADRB index stratification was determined by use of Akaike's information criterion (AIC).

Multivariate analyses used the following strategy for model selection: (i) A model containing all variables with a *P* value≤0.3 was first fitted. (ii) Variables were then excluded according to a backward elimination procedure. (iii) Variables with dichotomized *P* values>0.3 were added to the model, one by one, and any variable that reduced the deviance significantly was retained in the model. (iv) Interactions among variables were determined for inclusion in the final model.

Statistical analyses were performed with Egret (version 3.01; Cytel) and Statview (version 5.0; SAS Institute) software.

## Results

### Merozoite preparations

**Figure 1:**
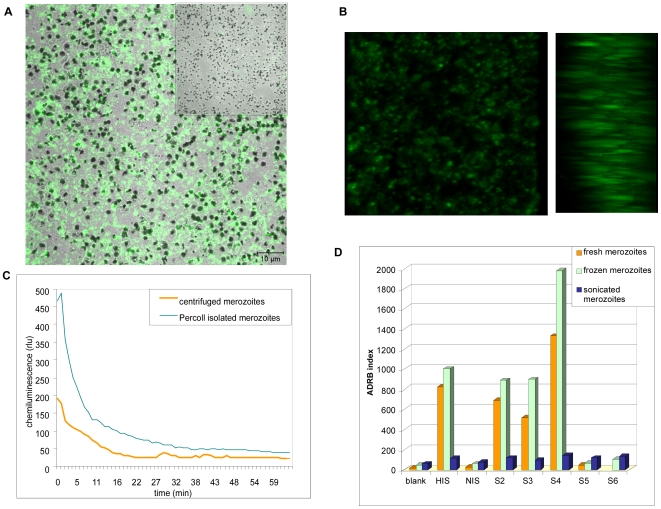
Characterization and activity of merozoite preparations. (A) Confocal imaging of a merozoite preparation showing a simple transmission image superimposed with immuno-fluorescence staining by a 1∶500 dilution of rabbit polyclonal anti-baculovirus recombinant PfMSP1p19 [35], and a 1∶1000 dilution of Alexa Fluor 488 conjugated goat anti-rabbit IgG (H+L) (Molecular Probes). The insert at upper right shows control immuno-fluorescence staining on the same preparation using a 1∶500 dilution of rabbit polyclonal anti-[baculovirus-null infected insect cells] and the Alexa Fluor 488 conjugate. (B) Immuno-fluorescence staining with anti-PfMSP1p19 as in (A) above showing two images from a confocal 3-D projection, either a direct view perpendicular to the slide (left) or at a 90° angle (right). The images are enlarged 5x compared to (A). (C) Chemiluminescence using HIS and merozoites prepared from (i) synchronized mature schizont cultures and percoll fractionation (blue trace) or (ii) centrifuged culture supernatants (orange trace). (D) ADRB indexes determined with a single pool of PMN using fresh (orange), frozen (green) or sonicated (3×5″ at 80 watts) (blue) merozoites and immune (HIS, S2, S3, or S4) or non-immune (NIS, S5, S6) sera.

Since merozoites are the key inducers of ADRB activity, reproducible high throughput chemiluminescence analyses required access to ample supplies of suitable merozoite material. Two protocols were investigated for merozoite preparation. (i) Large quantities of merozoites could be regularly harvested from post-invasion media of cultures with 5–10% parasitemia by centrifugation, but the preparations included some contaminating debris. Figure 1A shows a preparation using this method. Merozoites are visualized by confocal micrography as dense particles of 1–2 µm which are labeled by green immunofluorescence staining using a rabbit polyclonal antiserum raised to baculovirus recombinant *P. falciparum* MSP1p19. The insert at upper right shows immunofluorescence results using a control rabbit antiserum raised to insect cells infected with a baculovirus-null construct expressing no heterologous protein, indicating that the anti-MSP1p19 staining is highly specific and not due to possible contaminants present in the immunizing MSP1p19 antigen preparation. Diffuse light staining in Figure 1A is due to merozoites out of focus in the field shown. This can be seen in Figure 1B showing two images at a 90° angle of a confocal 3-D projection indicating that direct visualization of anti-MSP1p19 immunofluorescence staining at left gives a poor representation of what is actually a dense layer of merozoites that are not all in the same plane (right). The elongated aspect of the individual merozoites is an artifact of the confocal imaging technioque. (ii) Percoll gradient extraction from synchronized cultures of mature schizonts gave cleaner, more concentrated merozoites, and the best chemiluminescence (Figure 1C). However, this technique yielded limited material and damaged cultures, so it was unsuitable for high throughput. Freezing merozoite pellets, increased ADRB activity somewhat (Figure 1D), but antibody complexes with putative cytosolic antigens did not explain the increase, since merozoite sonication abrogated most ADRB activity (Figure 1D). Importantly, this result confirmed that opsonized merozoites, even if permeabilized by freezing, mediate ADRB activity, and was a key condition for the technical feasibility of the assay. In all subsequent experiments centrifuged frozen merozoites were used.

### Effector cells

The best PMN preparations regarding both yield and activity were obtained using a single step ficoll gradient separation followed by RBC lysis. Donor dependant variability in PMN activity was minimized by using pools from several donors, and also allowed more sera to be tested in parallel. PMN from non-infected European or Senegalese donors were equally reactive with immune sera from both sites (data not shown). However, the requirement for fresh PMN and utilization on the day of harvest is the *sine qua non* of this assay. In this study PMNs were used as rapidly as possible after harvest and purification. However, it was determined subsequently that higher chemiluminescence signals can be obtained when PMNs remain at room temperature 3–4 hr after harvest before purification (but not overnight), possibly due to up-regulation of Fc receptors.

### PMN respiratory burst activity depends on merozoites and immune antibodies

**Figure 2:**
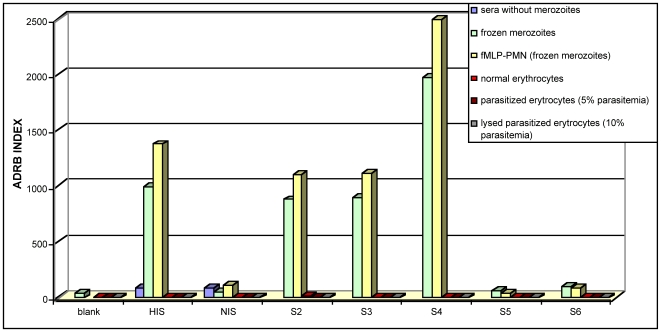
PMN respiratory burst activity depends on merozoites and immune antibodies. ADRB indexes determined as described in Materials and Methods using a single pool of PMN: with immune (HIS) or non-immune (NIS) sera only (blue); with merozoites and immune (HIS, S2, S3, or S4) or non-immune (NIS, S5, S6) sera (green) or the same with fMLP activated PMN (yellow); with normal (light red), parasitized (dark red) RBC, or lysed parasitized RBC (3x freeze-thawed) (grey) in the presence of immune or non-immune sera.

To confirm that PMN respiratory bursts are induced only by merozoites [26], [40], chemiluminescence was measured in the presence of immune serum using: (i) merozoites, (ii) normal RBC, (iii) RBC with 5% parasitemia (>80% schizonts and rosettes), (iv) lysed parasitized RBC (10% parasitemia) or (v) neither merozoites nor erythrocytes. Figure 2 shows that PMN ADRB activity depends exclusively on merozoites, and not on possible contaminants present in merozoite preparations, including normal or parasitized RBC, lysed parasitized RBC (containing hemozoin, food vacuoles, membrane debris etc.), or on immune sera in the absence of merozoites. Thus, any contaminants in the centrifuged merozoite preparations as seen in Figure 1A, would not contribute to chemiluminescence readout. Strong chemiluminescence was observed only in the presence of PMN, merozoites (fresh or frozen) and immune sera together. Neither PMN alone, with or without HIS, nor PMN and merozoites, with or without non-immune sera, could elicit chemiluminescence above background levels. PMNs previously activated with fMLP showed increased chemiluminescence, indicating that internal positive control values do not represent the maximal response detected by the system, also confirmed by S4 results in Figure 2, with ADRB index values almost twice as high as the HIS controls.

### Standardization of chemiluminescence readout

**Figure 3:**
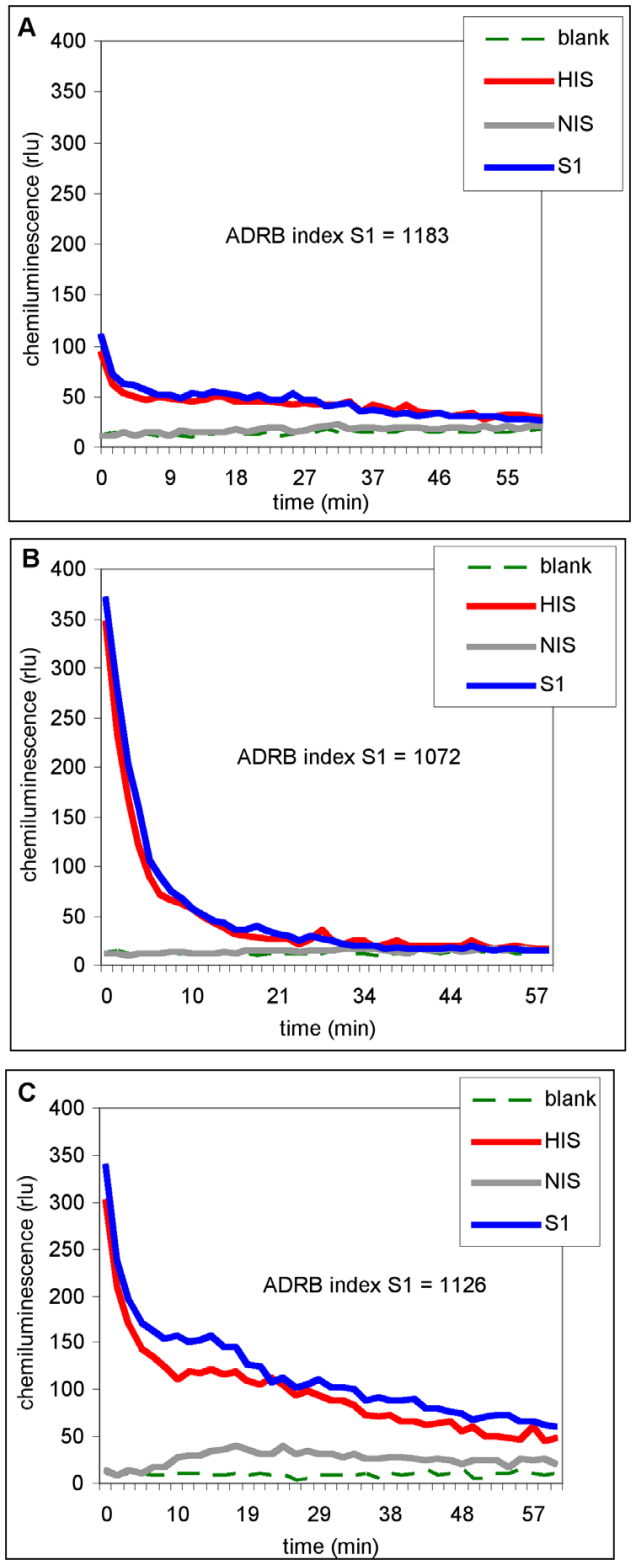
ADRB chemiluminescence profiles can be standardized. (A-C) Chemiluminescence profiles from 3 different ADRB experiments (3 PMN pools) tested without serum (blank, green trace), or in the presence of NIS (grey trace), HIS (red trace) and the same individual sample serum (S1, blue trace). The ADRB index for S1, calculated as described in Materials and Methods using HIS as a positive internal standard, is shown for each experiment.

Figure 3 shows chemiluminescence readout as a function of time from three independent experiments (three different PMN pools) either without serum (blank), or with NIS or HIS sera pools or serum from a single immune donor (S1) (Figure 3A-C). NIS alone showed only background reactivity similar to readouts without serum. Although the curves are different in the three experiments, those generated by the S1 sample and the HIS standard in the same experiment are very similar. In particular, in each case the S1 maximal rlu is reproducibly slightly higher than the HIS, even though the absolute values for both are different for each experiment. Thus, using maximal HIS rlu values as an internal standard, a reproducible arbitrary unit, the ADRB index, could be calculated as described in Materials and Methods. ADRB indexes determined for the S1 sample in the three experiments shown in Figure 3 were 1183, 1072 and 1126, yielding a mean of 1127+/−5%. Generally, intra-assay reproducibility (1 PMN pool) was >95 %, while inter-assay reproducibility (different PMN pools) was >80 %. It is possible that the area under the curves could be used as the readout rather than maximal rlu, since the curves were similar within a given experiment (same PMN batch), but this is a much more complicated measure, likely reflecting more complex phenomena. The standardization using maximal rlu was another sine qua non of the experimental approach, allowing data from different experiments to be compared.

### Dependence of chemiluminescence activity on IgG and complement

**Figure 4:**
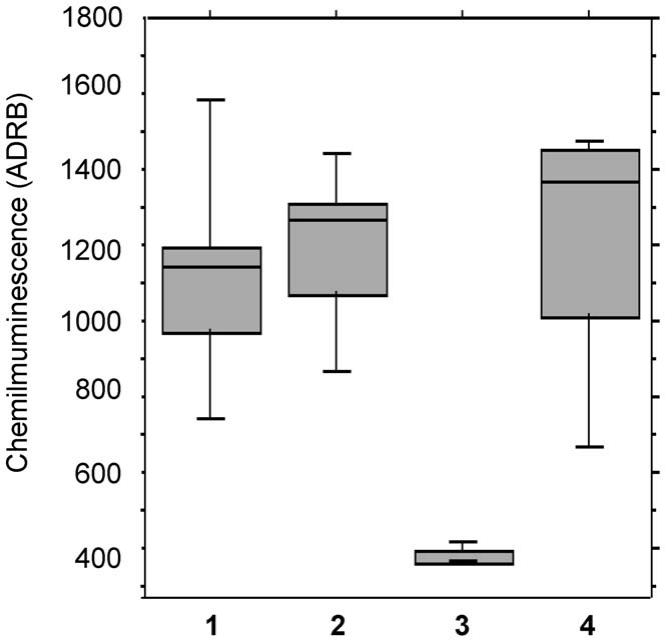
Serum IgG, but not complement, mediates ADRB activity. ADRB indexes were determined individually using 7 different immune sera either untreated (lane 1), after inactivation of complement (lane 2), after IgG depletion (lane 3), or after purification of IgG (lane 4). Distribution and median values are indicated by vertical and horizontal lines respectively.

To investigate the role of IgG in mediating chemiluminescence activity, seven sera with high ADRB values were individually depleted of IgG by passage over Protein-G, and the flow-through was concentrated to the original volume (IgG depleted serum). Figure 4 shows that: (i) untreated sera or after complement inactivation (lanes 1 and 2 respectively) induce statistically similar chemiluminescence signals using the Wilcoxon signed rank test (*P* = 0.5), (ii) purified IgG (lane 4) induces chemiluminescence signals similar to untreated sera (*P* = 0.4) or after complement inactivation (*P* = 0.6), and (iii) sera depleted of IgG gave negligible activity (lane 3). These results demonstrated the IgG dependence of ROS release, and indicated that there was no essential requirement for complement under the conditions of the ADRB assay.

### Comparison of ADRB activity in meso or holo-endemic conditions

ADRB indexes were compared in sera from residents of Ndiop (114) and Dielmo (116), corresponding to meso- or holo-endemic settings respectively. The median ADRB index for Dielmo was significantly higher than for Ndiop (268 and 179 respectively, *P* = 0.002). This likely reflects higher anti-merozoite antibody levels due to the prevailing holoendemic conditions.

**Figure 5:**
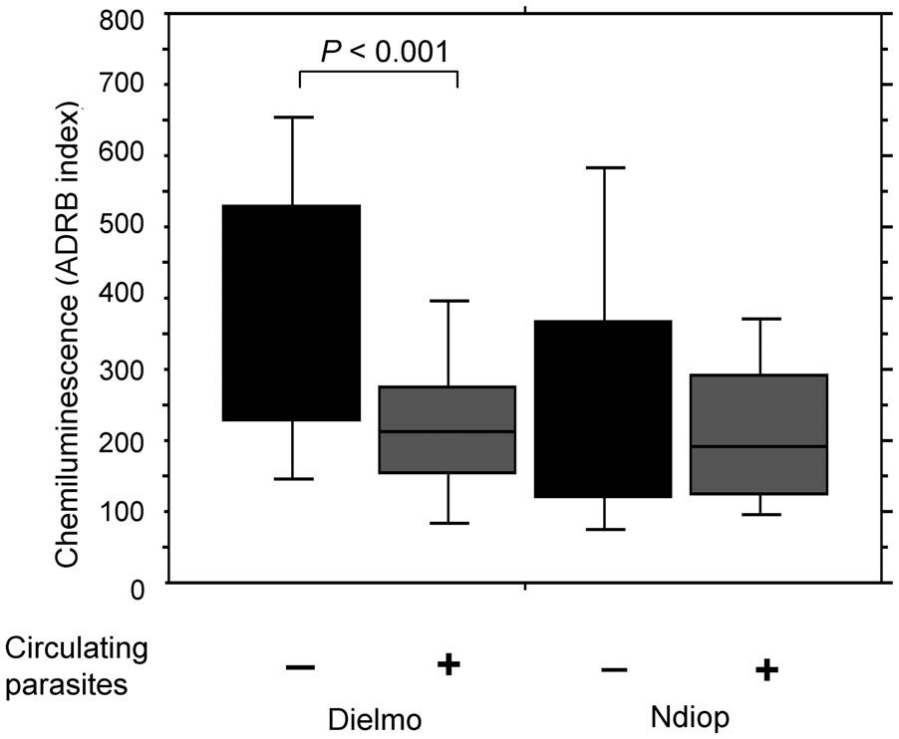
Comparison of ADRB index profiles for Dielmo and Ndiop. ADRB index values for Dielmo and Ndiop were analyzed as a function of parasite carriage at the time of sampling. Statistical significance was determined using the Mann-Whitney test and significant *P* values are noted.

When ADRB indexes from the Dielmo cohort were compared between the group of apparently healthy individuals, who nevertheless had circulating parasites at the time of sampling (58 asymptomatic carriers), and the group without circulating parasites (58 individuals), an average 36% reduction was observed in the former (*P*<0.001, Figure 5). A similar trend was observed in Ndiop, as indicated by 75 percentile levels (top of bars). However, since there were only 17 asymptomatic carriers (22%), the data did not reach statistical significance. These results could be explained by hypothesizing that parasitemia is being controlled to relatively low levels in asymptomatic carriers in part by anti-MSP antibodies which bind to merozoites, reducing the amounts of free anti-MSP antibodies available to mediate ADRB activity in serum samples from such individuals.

### ADRB activity is correlated with anti-merozoite cytophilic IgG1 and IgG3 antibody titers

**Figure 6:**
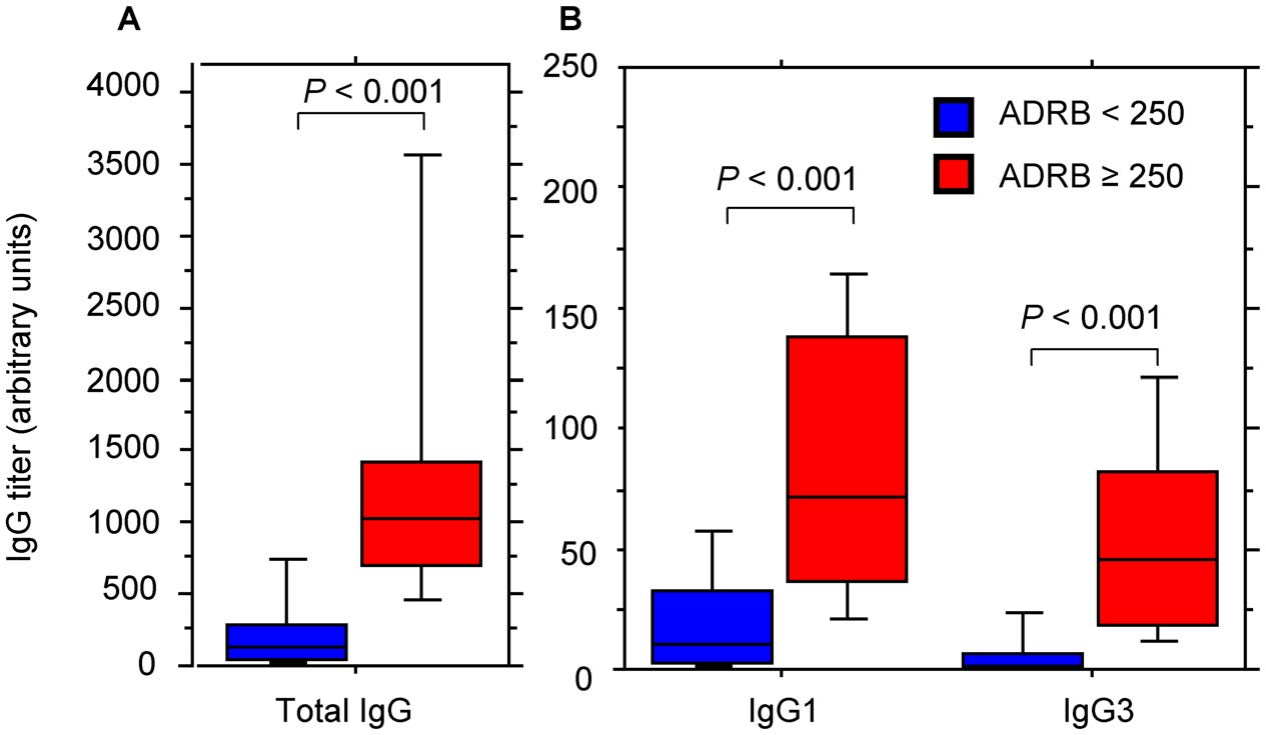
Chemiluminescence activity correlates with anti-merozoite IgG1 and IgG3 isotype titers. Sera from 88 aparasitemic Ndiop residents were stratified into 2 groups with ADRB indexes <250 (blue, 59 sera) or ADRB ≥250 (red, 29 sera). Stratified ADRB indexes are shown as a function of (A) total anti-merozoite IgG titer (1∶5000 dilution), and (B) separate anti-merozoite IgG1 and IgG3 titers (1∶200 dilution for both). Statistically significant differences, as detected with the Mann-Whitney test, are indicated.

To determine if ADRB activity is associated with particular IgG isotypes, sera from 88 aparasitemic Ndiop residents were monitored for merozoite antibodies using isotype specific reagents. While only 3 or 4 sera were above background for IgG2 and IgG4 respectively (dilution 1∶100), all were positive for IgG1 and IgG3. Arbitrary total IgG, IgG1 and IgG3 titers were calculated for each serum using the HIS standard regression curve. When ADRB indexes were plotted against anti-merozoite IgG titers (IgG total, IgG1 or IgG3), there was a correlation, but the coefficient of correlation R^2^ was very low, ranging from 0.14 to 0.44 and the linear or log-linear plots weren't very convincing (data not shown). For this reason we preferred to consider ADRB versus IgG associations as a function of the dichotomized ADRB levels, which were the same in both settings, and which appear to have clinical relevance (see below). Figure 6 shows that anti-merozoite IgG, IgG1 and IgG3 titers correlated positively with ADRB indexes (*P*<0.001 for all, Mann-Whitney test). To implicate preferentially IgG1 or IgG3 in ADRB activity, the Spearman test was used to compare IgG3:IgG1 ratios stratified into three groups: (i) <0.5 (ii) ≥0.5<1 and (iii) ≥1 with unstratified ADRB indexes. A significant positive correlation was seen only for sera with an IgG3:IgG1 ratio of <0.5 (*P* = 0.002), suggesting there may be a predominate role for IgG1.

### Association between ADRB index and susceptibility to clinical malaria at a meso-endemic site

**Figure 7:**
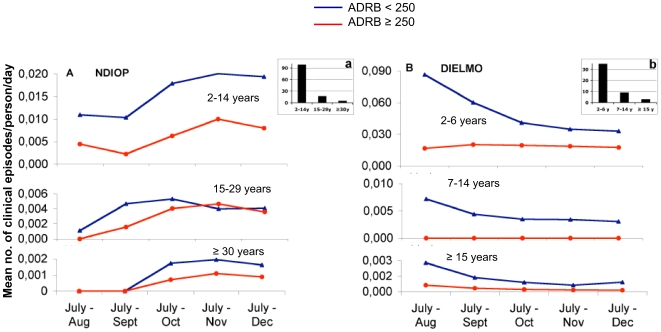
Dichotomized ADRB indexes have an age-dependent impact on clinical episodes at both low and high transmission sites. Analyses of associations between ADRB index and the number of clinical episodes experienced during a 5.5-month follow-up period, gave the best Poisson regression model with a cut-off of 250. The cumulative mean no. of clinical episodes/person/day at monthly intervals across the follow-up period were plotted for different age groups as a function of ADRB index <250 (blue trace) or ≥250 (red trace). (A) Ndiop: age groups: <15 years, 15–29 years, and ≥30 years (Table 1); (B) Dielmo: age groups: <7 years, 7–14 years, ≥15 years (Table 3).

Associations were analyzed between ADRB indexes and the number of cumulative clinical episodes experienced during the 5.5-month 2002 transmission season in Ndiop (meso-endemic). The definition of malaria episodes, the calculation of individual time at risk, and the basis of age brackets for malaria susceptibility in Ndiop are described in Materials and Methods. The best Poisson regression model according to AIC was obtained using a cut-off close to the mean ADRB index value (250). A bivariate analysis showed that the variables significantly associated with the number of clinical attacks were (i) the continuous ADRB index ([RR] = 1.004 per ADRB unit lost, *P*<0.001), or the dichotomized ADRB index, (ii) age, and (iii) hemoglobin phenotype (normal vs AS) (Table 1). Conversely, positive parasitemia at enrolment was not associated with the number of clinical attacks. Following multivariate analysis described in Materials and Methods, two alternative models were proposed: (i) using continuously decreasing ADRB index (RR = 1.002, *P* = 0.049), age (15–29 vs. ≥30 years, RR^1^ = 3.3; 2–14 vs ≥30 years, RR^2^ = 12.2; *P* = 0.02 and *P*<0.001, respectively), and hemoglobin phenotype, or (ii) dichotomized ADRB index, age, and hemoglobin phenotype (Table 1). Hemoglobin phenotype was no longer significant in either model. ADRB index and age were still significantly associated with immune status when using the mean ADRB index cut-off of 250 as shown in Table 1. The second model was the best (AIC = 120.4 vs 120.6) indicating that the risk of malaria attack was nearly 1.8-fold higher overall for individuals with ADRB index <250 than for those with ADRB index ≥250, after adjustment for age (*P* = 0.036, Table 1). The impact of ADRB index dichotomization at 250 with regard to cumulative clinical episodes, recorded over the 5.5 month follow-up period in different age groups (inversely related), is shown in Figure 7A. Within each age group, individuals with a serum ADRB index ≥250 before the transmission season had systematically fewer clinical episodes than those with ADRB index <250, except at the end of the transmission season in the 15–29 year group. This association between ADRB index and morbidity was most striking for the most susceptible 2–14 year olds.

**Table 1:** Poisson regression analysis of ADRB index values and clinical outcome for Ndiop.

NDIOP		Bivariate analysis			Multivariate model		
Category	Subcategory	RR	95%CI	*P*	RR	95%CI	*P*
**ADRB**	≥250	**1**			**1**		
	<250	**3.673**	2.171–6.215	<0.001	**1.789**	1.038–3.077	0.036
**Age (years)**	≥30	**1**			**1**		(<0.001)
	15–29	**3.632**	1.349–9.783	0.01	**3.203**	1.182–8.681	0.022
	2–14	**15.925**	6.484–39.116	<0.001	**12.627**	5.036–31.660	<0.001
**Hemoglobin phenotype**	AA	**1**					
	AS	**3.310**	1.616–6.780	0.001			NS
**Deviance**					**118.4**		
**AIC**					**120.4**		

Bivariate and multivariate Poisson regression models were built to analyze the relation between ADRB and acute malaria morbidity. The bivariate model includes morbidity and analysis of one variable. The multivariate model includes morbidity and other variables such as age and dichotomized ADRB index (above and below the mean ADRB value of 250), where the impact of the different variables is analyzed together. The *P* value in parenthesis refers to the overall impact of age on the multivariate model. (NS: non significant.)

Anti-merozoite IgG levels and clinical outcome for non-parasitized Ndiop inhabitants were correlated after bivariate analysis (*P*<0.001), but anti-merozoite IgG was no longer significant when age was included in the Poisson regression analysis for the multivariate model (*P* = 0.91, Table 2).

**Table 2:** Poisson regression analysis of anti-merozoite IgG levels and clinical outcome for non-parasitized Ndiop inhabitants.

NDIOP		Bivariate analysis			Multivariate model		
Category	Subcategory	RR	95%CI	*P*	RR	95%CI	*P*
**anti-merozoite IgG level**	(continous)	**0.999**	0.9985–0.9995	<0.001	**1.000**	0.9996–1.0004	NS
**Age (years)**	≥30	**1**			**1**		(<0.001)
	15–29	**3.632**	1.349–9.783	0.01	**2.446**	0.822–7.271	NS
	2–14	**15.925**	6.484–39.116	<0.001	**16.432**	5.954–45.350	<0.001
**Deviance**					**87.8**		
**AIC**					**89.8**		

Bivariate and multivariate Poisson regression models were built to analyze the relation between anti-merozoite IgG levels and acute malaria morbidity. The bivariate model includes morbidity and analysis of one variable. The multivariate model includes morbidity and other variables such as age and IgG levels, where the impact of the different variables is analyzed together. The *P* value in parenthesis refers to the overall impact of age on the multivariate model. (NS: non significant.)

### Association between ADRB index and susceptibility to clinical malaria at a holo-endemic site

Similar associations between ADRB index and number of cumulative clinical episodes, experienced during the same 5.5 month follow-up period in 2002, were analyzed in Dielmo (holo-endemic). Analyses were carried out as described above for Ndiop. As for Ndiop, a bivariate analysis showed that the variables significantly associated with the number of clinical attacks were (i) the continuous and dichotomized ADRB index (continuous rate ratio [RR] = 1.001 per unit lost; dichotomized rate ratio at 300 (mean value) RR = 30.6; dichotomized rate ratio at 250 (Ndiop model value) RR = 11.8; *P*<0.001 for all), and (ii) age (values in Table 3). Conversely, hemoglobin phenotype and positive parasitemia at enrolment were not associated with the number of clinical attacks, although hemoglobin phenotype had a significant effect in some multivariate models. After multivariate analysis, three alternative models were proposed using: (i) continuously decreasing ADRB index (RR = 1.005, *P* = 0.006) and age (7–14 vs. ≥15 years RR^1^ = 4.2; 2–6 vs. ≥15 years RR^2^ = 31.9; *P* = 0.032 and *P*<0.001 respectively), (ii) ADRB index dichotomized at 300 units (<300 vs ≥300, RR = 12.2, *P* = 0.014), and age (7–14 vs. ≥15 years RR^1^ = 3.8; 2–6 vs. ≥15 years RR^2^ = 41.1; *P* = 0.046 and *P*<0.001 respectively) and (iii) ADRB index dichotomized at 250 units, age, and hemoglobin phenotype (Table 3). ADRB index and age were still significantly associated with immune status for each model. The third model (Table 3), using the same cut-off ADRB value of 250 as for Ndiop, was the best (AIC = 90.7 vs 113.6 and 108.6). In this model hemoglobin phenotype was not significant, but statistically important (score test Hb: *P* = 0.035). Strikingly, the model predicts that in this holo-endemic setting the risk of malaria attack is over 17-fold higher for individuals with an ADRB index <250 compared to those with an ADRB index ≥250 after adjustment for age, equivalent to a 94% reduction of clinical episodes (*P* = 0.006). As above, the impact of functional antibodies in the ADRB assay with regard to cumulative episodes in different age groups is shown in Figure 7B. Again, regardless of age group, individuals with a serum ADRB index ≥250 had systematically fewer clinical episodes than those with ADRB index <250, especially in the younger, most susceptible age groups. The use of different scales of malaria incidence in Figure 7 is a reflection of both different transmission rates and age dependent susceptibility.

**Table 3:** Poisson regression analysis of ADRB index values and clinical outcome for Dielmo.

DIELMO		Bivariate analysis			Multivariate model		
Category	Subcategory	RR	95%CI	*P*	RR	95%CI	*P*
**ADRB**	≥250	**1**			**1**		
	<250	**11.762**	4.669–29.631	<0.001	**17.509**	2.275–134.744	0.006
**Age (years)**	≥15	**1**			**1**		(<0.001)
	7–14	**8.426**	2.595–27.362	<0.001	**2.876**	0.759–10.904	NS
	2–6	**87.542**	31.244–245.282	<0.001	**17.637**	5.042–61.690	<0.001
**Hemoglobin phenotype**	AA				**1**		
	AS				**0.79**	0.291–2.141	NS
	Scoretest Hb			NS			0.035
**Deviance**					**86.7**		
**AIC**					**90.7**		

Bivariate and multivariate Poisson regression models were built to analyze the relation between ADRB and acute malaria morbidity. The bivariate model includes morbidity and analysis of one variable. The multivariate model includes morbidity and other variables such as age, ADRB index dichotomized at 250, and hemoglobin phenotype, where the impact of the different variables is analyzed together. The *P* value in parenthesis refers to the overall impact of age on the multivariate model. (NS: non significant.).

## Discussion

Recent years have seen significantly increased attention focused on the search for malaria vaccines offering broad, long-lasting protection. Many vaccine candidates have been proposed, but the high cost of clinical testing has underscored a need for prioritization, and led to persistent calls for development of *in vitro* functional tests correlated with immune protection in humans. The latter parameter implies either monitoring infection after deliberate challenge, which is ethically controversial for blood stage candidates, or longitudinal monitoring of clinical episodes in endemic populations. In both cases “correlates” implies large enough sampling to ensure statistical relevance. Some studies have correlated specific antibody levels with clinical protection [2]–[5], but not functional attributes of these antibodies. Conversely, while ADRB functional IgG activity was highly correlated with clinical outcome here (especially for Dielmo and for the youngest children in Ndiop), anti-merozoite IgG levels (ELISA) were not when age was also considered, showing that ADRB functionality was a more relevant predictor of clinical outcome.

These results constitute the first clear demonstration of a functional antibody immune correlate of protection against *P. falciparum* malaria pathology in humans in two different transmission settings. The approach used relied on two essential elements: (i) development of a reproducible standardized, high-throughput assay quantifying antibody dependent respiratory burst (ADRB) activity by PMN, and (ii) access to anti-sera from a large cohort of endemic donors participating in a cross sectional prospective study with intensive clinical follow-up. The standardized protocol permitted inter-experimental comparisons, thereby increasing the statistical power to investigate associations between the functional activity of antibodies induced by natural exposure and clinical protection. Extensive epidemiological data have been collected for more than 15 years from Ndiop and Dielmo with seasonal or year round transmission respectively. Data from both settings were analyzed in an age-adjusted Poisson regression model, and showed that donors with serum ADRB indices ≥250, had significantly fewer clinical malaria episodes than those with ADRB <250, translating into a striking 17-fold risk factor associated with this parameter in Dielmo, and a 1.8-fold risk factor in Ndiop. The difference in risk factors between the two sites has both statistical and physiological explanations. Statistically it is linked to the fact that in Dielmo, only 22 of 116 villagers experienced one or more malaria episodes, whereas in Ndiop there were 3 times as many having clinical accesses (63/114). But 20 of the 22 Dielmo villagers with malaria had an ADRB index < 250, compared to 51 of 63 in Ndiop, explaining both the stronger RR value and the larger confidence interval in the former. On a physiological level it is notable that Dielmo has an infection pressure (number of infectious bites) over 10 times stronger than Ndiop, although Dielmo villagers have 2–3 fold fewer episodes. Thus factors related to clinical protection are more intensively “boosted” in Dielmo resulting in much stronger associations.

It is notable here that the clinically relevant dichotomized value of 250 for ADRB index was independently found to be the same in both villages in spite of the very different transmission characteristics. In addition the 250 value was the mean ADRB index in Ndiop and close to the mean (300) in Dielmo. Thus, there may be a “threshold” ADRB response important for clinical protection, even if the precise biological phenomena it reflects are not clear.

Taken at face value, these results suggest, but do not prove, that extra-cellular ROS release induced in PMNs by opsonized merozoites, may be a key effector function for protection against blood stage *P. falciparum* infection, despite the lack of prominence of either PMN or ROS in many reviews on the subject, if they are mentioned at all [20], [21]. Several observations are consistent with this hypothesis. (i) PMNs have generally shown the most intense respiratory burst activity when stimulated by *P. falciparum* asexual blood stage parasites, particularly merozoites [12], [27], [41] and PMN growth inhibition of *P. falciparum in vitro* was substantially dependent on antibody opsonins in immune serum [42]. (ii) The isoluminol reagent used here detects specifically extra-cellular ROS metabolites [23], which are known to be particularly toxic for intra-erythrocytic parasites [17], [18], [19]. ROS could be a more effective agent targeting this form of the parasite than specific antibodies, given the extreme polymorphism of parasite antigens on the surface of the infected red cell. (iii) ADRB activity apparently did not require prior PMN activation, since freshly harvested cells from normal donors released ROS instantaneously upon contact with opsonized merozoites, suggesting that circulating PMNs would be rapidly responsive to the presence of this stimulus *in vivo*. (iv) Neutrophils represent 50–75% of blood leucocytes, and undergo very characteristic changes in patients infected with *P. falciparum* (or *P. vivax*) [43], where neutropenia is frequent and neutrophil populations often shift towards more immature forms. (v) ROS mediated killing of intra-erythrocytic parasites could explain the observation that concurrent infections by *P. falciparum* and *P. vivax* are mutually suppressive [44], since PMN generation of ROS, induced by opsonized merozoites of either species, would be equally effective against intracellular parasites of both species. Nevertheless, a correlation between children with severe malarial anemia and enhanced innate production of ROS by granulocytes, is indicative of a possible negative effect of ROS on the host [45].

The similarities of the ADRB and ADCI assays suggest that they may actually reflect the same phenomena. The short half-life of PMNs compared to monocytes, could explain why the former did not appear to be functional during the long 24–96 hr culture periods required for the ADCI assay [12], [46]. Notably, we observed a rapid decline in the capacity of freshly harvested PMNs to generate ROS, even over the short 60 min duration of the ADRB assay. Since activated monocytes (and macrophages) also release ROS, this could be the unidentified soluble factor mediating ADCI activity. In addition, the prolonged *in vitro* ADCI assay conditions are likely to transform and activate monocytes in ways not relevant to their *in vivo* function as a relatively small inactive pool of macrophage precursors in the blood. Singlet oxygen may preferentially mediate ROS growth inhibition of *P. falciparum*, explaining the inability of superoxide dismutase and catalase to block ADCI activity [12], [46].

Several important questions regarding the molecular basis of ADRB activity remain to be answered. (i) It was dependent on serum IgG1 and IgG3 isotypes, which mediate antibody interactions with accessory cells via Fcγ receptors, and are associated with clinical protection against malaria [47], [48]. Cross-linking of high affinity FcγRI triggers PMN respiratory bursts, but FcγRI expression is inducible in PMNs, and could be up-regulated by cytokines such as TNFα, IFNγ and TNFβ, which increase antibody-dependent chemiluminescence signals [26]. Although the PMN were from healthy donors and used within hours of harvest, we do not know what Fcγ receptors were expressed. Nevertheless, the primary limitation of the ADRB assay is linked to the characteristic fragility of PMNs, which have a very short half-life *in vivo* (around 7 hr when circulating). (ii) The ADRB assay requires antibodies compatible with human Fcγ receptors, so those induced by vaccination of animals may not be functional. Although antibodies to *P. falciparum* MSPs induced in rabbits were not functional, those raised in at least some primate species may be compatible. (iii) Since the toxicity of ROS generated by PMN is postulated to be an important factor in ADRB association with protection, the inhibitory effects of ADRB assay supernatants on *P. falciparum* cultures should be evaluated, and the putative implication of particular ROS determined using reversible inhibitors such as histidine, tryptophan, superoxide dismutase and/or catalase, to indicate which oxygen radicals, if any, are involved [49]. (iv) ROS release has often been considered to be primarily a reflection of PMN phagocytosis of opsonized merozoites, but quantitative links between the two processes are not clear. The use of a flow cytometric phagocytosis assay in association with ADRB readouts could help to establish a clearer relationship [50]. Further work should attempt to address the issues outlined here.

In related work, the ADRB assay was used to evaluate the functionality of antibodies in human immune sera specific for baculovirus PfMSP1p19, PfMSP4p20 and PfMSP5 vaccine candidates, and has provided strong arguments for the vaccine candidacy of some of these antigens (manuscript in preparation). If reproducible in other laboratories, the ADRB assay could prove to be an important new tool for evaluating the functional relevance of merozoite specific antibodies induced either by natural infection or vaccination with recombinant merozoite vaccine candidates [8], [29]–[31].
